# Genomic and Non-Genomic Actions of Glucocorticoids on Adipose Tissue Lipid Metabolism

**DOI:** 10.3390/ijms22168503

**Published:** 2021-08-07

**Authors:** Negar Mir, Shannon A. Chin, Michael C. Riddell, Jacqueline L. Beaudry

**Affiliations:** 1 Department of Nutritional Sciences, Temerty Faculty of Medicine, University of Toronto, Toronto, ON M5S 1A8, Canada; negar.mir@mail.utoronto.ca (N.M.); Shannon.chin@mail.utoronto.ca (S.A.C.); 2 Faculty of Health, School of Kinesiology and Health Science, York University, Toronto, ON M3J 1P3, Canada; mriddell@yorku.ca

**Keywords:** lipolysis, adipose tissue, lipid metabolism, white adipose tissue, brown adipose tissue, glucocorticoid, corticosterone, dexamethasone, metabolic disorder, diabetes, obesity, glucocorticoid receptor, genomic mechanism, non-genomic mechanism

## Abstract

Glucocorticoids (GCs) are hormones that aid the body under stress by regulating glucose and free fatty acids. GCs maintain energy homeostasis in multiple tissues, including those in the liver and skeletal muscle, white adipose tissue (WAT), and brown adipose tissue (BAT). WAT stores energy as triglycerides, while BAT uses fatty acids for heat generation. The multiple genomic and non-genomic pathways in GC signaling vary with exposure duration, location (adipose tissue depot), and species. Genomic effects occur directly through the cytosolic GC receptor (GR), regulating the expression of proteins related to lipid metabolism, such as ATGL and HSL. Non-genomic effects act through mechanisms often independent of the cytosolic GR and happen shortly after GC exposure. Studying the effects of GCs on adipose tissue breakdown and generation (lipolysis and adipogenesis) leads to insights for treatment of adipose-related diseases, such as obesity, coronary disease, and cancer, but has led to controversy among researchers, largely due to the complexity of the process. This paper reviews the recent literature on the genomic and non-genomic effects of GCs on WAT and BAT lipolysis and proposes research to address the many gaps in knowledge related to GC activity and its effects on disease.

## 1. Introduction 

Glucocorticoids (GCs) are potent regulators of whole-body energy metabolism, elevating circulating glucose and free fatty acid (FFA) levels in times of stress. They are steroid hormones that include active cortisol (human) and corticosterone (CORT; rodent) hormones and their inactive counterparts, cortisone and 11-dehydrocorticosterone [1]. Dexamethasone (DEX) is a potent synthetic GC used as an anti-inflammatory medication and for studying the actions of GCs on metabolism and immune function [2]. The advent of the SARS-CoV-2 (COVID-19) disease has led to an increase in emergency DEX use in an effort to decrease mortality in patients on respiratory support [3].

The body synthesizes endogenous GCs, releasing them from the adrenal cortex to act on multiple tissues, including adipose tissue (AT), the liver, and the skeletal muscles [4]. In early development, GCs assist in lung maturation and function in the fetus, and, throughout adulthood, help regulate metabolism, inflammation, and the immune system [5]. By contrast, exogenous GCs given to assist fetal development may have detrimental effects on both mother and offspring, including changes to metabolism, fetal growth and development, disease risk, and stress reactivity [6,7,8]. There may also be adverse effects to the central nervous system, with changes in neuron length, number, or synapses, through the mechanism of structural plasticity [9].

Steroid- or chronic stress-induced increases in GC levels in the brain may result in behavioral and cognitive changes and induce mood disorders such as depression [10,11]. GCs may also modify food intake and preference for fatty foods, acting on multiple brain pathways involved in feeding and hunger [12]. Consequent increases in food intake may alter the level of lipids in the body and lead to AT accumulation. As mentioned above, GCs can work on multiple tissues in the body, including the AT. This review highlights the current state of knowledge of the powerful effects of GCs on lipid metabolism, and emphasizes AT lipolysis.

Genomic mechanisms act via the cytosolic GC receptor (GR) [4] and are ubiquitously expressed in several tissues in the body; these are the most studied activation mechanisms of GCs. The GR is mainly found in the cytosol, the intracellular fluid of the cell. After GCs bind to the GR, the GR translocates to the nucleus, binds to the GC response elements (GREs) on the DNA, and then works as a transcription factor influencing the expression of genes, corresponding to its effects on the tissue [13]. With different isoforms of GR and GREs varying among tissues, the metabolic and physiologic effects of GCs differ by tissue type. For example, the predominant GC signaling pathway in the liver increases hepatic glucose production, while its main effect in the AT is to regulate lipid metabolism [4]. The major GREs in the AT related to lipid metabolism include lipolytic genes (*Lipe* and *MgII),* triglyceride synthetic genes such as *Agpat2*, *Gpat3*, *Gpat4*, and *Lpin1*, genes related to lipid transport (*Cd36* and *Lrp1*), and genes related to lipid storage, such as S3-12 [14].

GCs can also bind to the mineralocorticoid receptor (MR), which has a 10-fold higher affinity than the GR, making the effects of GCs on MR more pronounced at basal levels of GCs [15]. MR activation increases in the visceral adipose of obese mouse models [16]; MR overexpression in mice adipocytes induces a phenotype resembling the metabolic syndrome. This involves increased fat mass and dyslipidemia [17], which increases the risk of coronary disease and stroke. In contrast, whole-body MR knockout mice have reduced fat mass and body weight [18]. It is estimated that there is sexual dimorphism in MR expression in humans, and its expression could be suppressed by the female biological clock [19]. However, more research is needed to determine how MR expression is affected by sex. In addition, in tissues such as the kidney, where there is an abundance of GC deactivation enzyme 11BHSD2, MR is predominantly bound to aldosterone instead of GCs. It is estimated that, in AT, a tissue with low levels of 11-BHSD2, GCs remain the main ligand of the MR [20]. The combined effects of GR-MR activation in response to GC is complicated and not completely understood. Our review will focus on the effects of GR activation in lipolysis, as the most often used compound in these studies, DEX, does not significantly activate the MR [21,22].

The non-genomic mechanisms of action of GCs can affect various tissue types, including those in the central nervous system and AT. In general, non-genomic actions (a) appear to happen within minutes after GC availability in the cytosol, (b) are usually insensitive to cytosolic GR blockade, and (c) are independent of nuclear gene transcription or translation [23,24]. Non-genomic pathways can influence a wide variety of cellular functions, such as regulating calcium ion transport or cell contraction, which appear to be dependent on the tissue type [25]. Non-genomic actions can also occur through translocation of the GR to the mitochondria, where it can bind to the mitochondrial chromosome and regulate genes related to energy metabolism [26]. The non-genomic effects of GCs in various tissue types, such as liver, skeletal muscle, and AT, have not been investigated as extensively as the genomic effects. However, non-genomic mechanisms represent diversifying modes of GC action that may help to explain how GCs influence tissue-specific metabolic pathways.

GC regulation is necessary for the normal functioning of the immune system and metabolism [27]. Even subtle disturbances in regulation have been linked to diseases such as diabetes [28], obesity [29], and a preference for higher fat density foods [30]. The endocrine disorder, Cushing’s syndrome, results from endogenous hypercortisolism, and usually manifests in multiple organs, with symptoms such as central obesity (accumulation of visceral fat in the abdominal area), hypertension, and muscle weakness [31]. Although this condition suggests simultaneous GC effects on multiple organs, the direct effects of GCs in AT and their relation to indirect impacts on multi-organ metabolic mechanisms is still unclear. The AT, however, remains a major site of GC action, largely regulating lipid metabolism through genomic and non-genomic signaling on several key features of adipogenesis, body adiposity, and lipolysis, as described below.

## 2. Adipose Tissue Biology

AT is a major organ for energy storage and release throughout the body. There are two major types of AT: the white adipose tissue (WAT) and the brown adipose tissue (BAT). WAT is the classic AT, its prominent function to store excess lipid energy in the form of triglycerides [32]. WAT is found in multiple locations, and includes subcutaneous adipose tissue (SAT), situated under the skin, and visceral adipose tissue (VAT), found next to vital internal organs [32]. It is generally held that SAT is a beneficial organ promoting insulation and vast energy stores, while VAT, if excessive, is associated with increased risk of cardiometabolic disease and most forms of cancer [33,34,35]. The amount of triglyceride stored in WAT can vary depending on the balance between lipogenesis (biosynthesis and storage of triglycerides) and lipolysis (hydrolysis of triglycerides) [36]. Lipolysis is predominantly regulated by lipase hormones, including adipose triglyceride lipase (ATGL) and hormone-sensitive lipase (HSL) in WAT [36]. Through genomic effects, GCs alter ATGL and HSL gene transcription and translation by binding to the GR in WAT, as evidenced in differentiated 3T3-L1 mouse derived cells [37]. GCs can exert non-genomic actions in the AT, though these effects, also leading to changes in lipolytic mechanisms [38], are rarely studied.

BAT is considered essential for protection from the cold, and is found in large quantities in smaller mammals and newborn babies and much smaller quantities in adult mammals [32]. Considered to have antagonistic effects to WAT functions, BAT’s excess energy is used for heat generation, through uncoupling respiration via the uncoupling protein 1 (UCP1), located along the inner mitochondrial membrane of BAT [32]. Due to its small quantity in adult mammals, BAT has been considered non-relevant to the energy process. Over the last decade, however, significant evidence that BAT activation or recruitment may help combat metabolic complications with diabetes and cardiovascular disease has increased interest in BAT’s role in whole-body metabolism and homeostasis in adult humans [39]. Studies indicate that GC exposure can result in increased lipid accumulation and decreased non-shivering thermogenesis in BAT [40], although the exact underlying mechanism is still unknown.

AT is a dynamic organ, where external and internal factors can lead to its remodeling. In response to diet, WAT can undergo hypertrophy, increased lipid droplet size, or hyperplasia, increased adipocyte numbers. Conversely, exercise and fasting conditions can reduce the depot area [41]. BAT can also adapt thermogenic activity according to the environmental cues, where warm or high fat diet conditions induce a white-like phenotype (whitening) [42]. Even though adaptability of AT can be beneficial, it can also be harmful and lead to AT malfunction. Abnormal fat collection in peripheral tissues develops due to dysregulation of fuel storage and utilization in AT, in response to exogenous and endogenous signals [43]. This ectopic fat accumulation, or obesity, is a leading risk factor driving the development of other comorbidities, such as insulin resistance and type 2 diabetes mellitus [26]. As AT plays a central role in metabolic and chronic disease development, understanding its physiology and hormonal balance is critical. As mentioned above, GCs play a major role in lipid metabolism in AT by altering protein expression or function, the exact mechanisms, however, remain unclear. Some literature suggests that GCs induce lipolysis in AT [44,45], while others indicate that GCs would accumulate and retain lipids in the AT [46], as well as increasing adipogenesis [47]. The discrepancies exist due to differences in the length of GC exposure, adipose depot and species investigated. For example, shorter exposure usually shows GC effects through non-genomic pathways, while chronic exposure results in recruitment of genomic pathways.

One of the most studied models of AT is the 3T3-L1 model. This immortalized cell line from 3T3 mouse embryos can be differentiated and acquire an adipocyte phenotype in the presence of an adipogenic cocktail, including 3-isobutyl-1-methylxanthine (IBMX), insulin, and DEX [48]. Another method of study of AT in vitro uses freshly isolated preadipocytes (primary AT) from animals that can also be differentiated into mature functioning adipocytes. Compared to the 3T3-L1 model, primary adipocytes are more difficult and expensive to culture and can be passaged fewer times. However, they provide more similarity to in vivo conditions, which makes them a valuable tool when studying the additive effects of multiple hormones and drugs on lipid metabolism [49]. The fully differentiated adipocytes can develop lipid droplets, and express major adipocyte transcription factors, such as peroxisome proliferator-activated receptor gamma (PPARγ). Adipocytes in the process of differentiating have a morphology and physiology that is in between preadipocytes and differentiated adipocytes [50,51]. The final stages of adipocyte differentiation require the PPARγ receptor. In addition to being the master regulator of adipocyte differentiation, it regulates genes involved in lipid metabolism in AT. Reports show that PPARγ is expressed more in males than in females [52], which could correspond to sex-dependent differences in male and female AT regulation [53].

There are several bodies of work that concentrate on distinguishing the genomic vs. non-genomic effects of GCs in peripheral tissues [54]. To date, there is limited empiric literature that focuses on the actions of GCs in AT, and that specifically addresses genomic and non-genomic pathways. The objective of this review paper is to compare the genomic versus non-genomic effects of GCs on lipid metabolism in AT, citing shortcomings and suggesting areas that need more investigation.

## 3. Genomic Effects of GCs on AT Lipolysis in WAT

**Figure 1:**
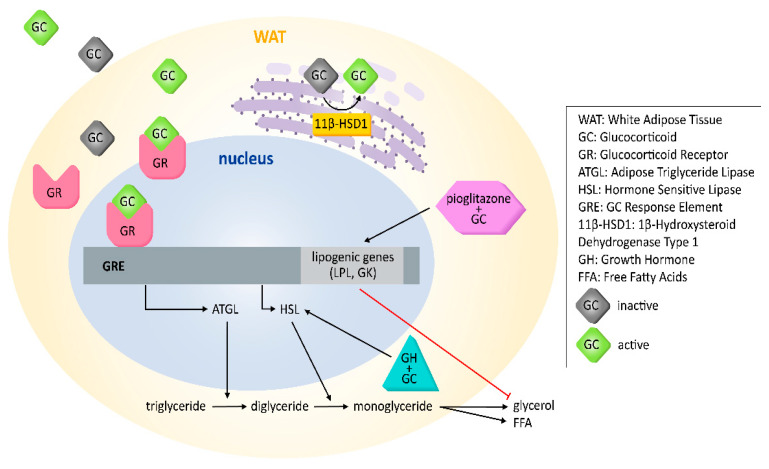
Genomic effects of GCs on lipolysis in WAT. The genomic effects occur through the binding of GC to the GR and the movement of GC-GR to the nucleus, where they can regulate the transcription of genes related to lipid metabolism, including lipolytic enzymes ATGL and HSL. Other regulators of lipid metabolism, such as pioglitazone (peroxisome proliferator-activated receptor gamma ligand) and growth hormone (GH), have counterbalancing effects to glycerol release that alter the way GCs induce lipolysis.

Genomic-induced pathways of GCs are dependent on GCs binding to the cytosolic GR. In WAT, the genomic actions usually alter the expression level of lipolytic enzymes ATGL and/or HSL. However, the exact result depends on duration of exposure, the GC level, the AT depot—such as SAT or VAT—and the presence or absence of other hormones. A summary of the genomic effects of GC on lipolysis in WAT is shown in Figure 1.

In a study by Hasan et al., incubation of DEX (0–100 nM) with 3T3-L1 differentiated cells for 6 days increased ATGL mRNA and protein levels, as well as lipolysis, measured by glycerol release in a dose-dependent manner; there were no effects on HSL mRNA or protein levels [55]. In a study by Slavin et al., they used differentiated rat primary epididymal adipocytes, and addition of DEX (0–100 nM) for 24 h led to a dose-dependent increase in *Lipe* (*hsl*) mRNA and glycerol release; these results were not seen within 4 h of exposure [56]. In another study by Xu et al., incubation of rat epididymal primary adipocytes with 1–1000 nM of DEX for 24 h led to increased glycerol release in a dose- and time-dependent manner [57]. The Xu study is one of the few studies in which the same experiment was repeated using DEX, cortisone, cortisol, and corticosterone, and where all these GCs led to a similar increase in FFA. However, glycerol concentrations were not reported for these four individual types of GCs. Glycerol is known to be a better marker of lipolysis than FFA, since it cannot be re-esterified and re-enter the tissue once it is secreted. Within the same study [57], the effects of DEX on lipolysis were blocked after co-incubation with RU486 (GR inhibitor) and actinomycin D (transcription inhibitor), showing the dependency of DEX genomic action on the GR. In summary, these limited investigations suggest that DEX exposure over a relatively short period (hours to days) can increase lipolysis in a dose- and time-dependent manner. However, whether DEX can affect ATGL, HSL, or both, appears to depend on the source of adipocyte in the experiment (3T3-L1 cells vs. primary adipocytes). The source of the adipocytes is vital in interpreting the results of each study, as different models of investigation can lead to vastly different data. It is also possible that DEX can increase HSL mRNA and protein levels in a 24h exposure, while not affecting expression after 24 h.

When DEX (1 mg/kg/d) was added for 12 weeks to the drinking water of mice, there were increases in lipolysis (glycerol release) as well as ATGL mRNA and protein level, with no changes in the HSL mRNA or protein levels. This increase was significantly exacerbated in diet-induced obese mice, indicating that obesity might synergically alter the effects of GCs on the body [58].

While increased GC exposure may increase lipase expression via potent and dose-dependent genomic actions, GCs may also be antilipolytic via their non-genomic actions [47].

## 4. Additional Genomic Effects of GCs on Lipolysis

GCs work alongside many other hormones and receptor ligands in the body that affect AT lipolysis. However, the effects of GCs on lipolysis are different depending on which pathways are activated. For example, even though DEX was shown to increase lipolysis [55] when differentiated 3T3-L1 cells were co-incubated with DEX and pioglitazone, pioglitazone decreased the DEX-induced lipolysis significantly. Pioglitazone is a PPARγ ligand. As the master regulator of adipocyte differentiation, PPARγ works as a transcription factor to alter genes involved in lipid metabolism [59,60]. Alone, pioglitazone enhances upregulation of ATGL and HSL protein, as well as some lipogenic gene expression levels. Together, DEX and pioglitazone increase lipolytic gene mRNA levels, but the enhancement of lipogenic genes by pioglitazone counterbalances these effects, and glycerol release decreases significantly. In addition, even though DEX alone does not affect HSL protein levels, it enhances the pioglitazone-induced increase and facilitates the pioglitazone-induced binding of HSL to PPARγ. This suggests permissive effects of GR activation on PPARγ activation and should be studied further [55]. We can conclude that the net effect of PPARγ activation and DEX decreases lipolysis and induces lipid accumulation, thereby potentiating problematic conditions of ectopic fat accumulation.

GCs have also been shown to interact with insulin in various aspects of lipid metabolism. For example, insulin decreases lipolysis in AT [61], probably through inhibition of the HSL protein [62]. GCs, on the other hand, act to increase *Lipe* mRNA, which can lead to an increase in lipolysis, hyperlipidemia in the blood, and insulin resistance [56]. This suggests that the effects of GCs on lipolysis might be dynamic, and depend on the presence of other stimuli, including insulin [63]. Since GCs can induce the secretion of insulin, this can work as a feedback loop to alleviate the negative, lipolytic effects of chronic GC secretion [64]. While GCs and insulin have opposing effects on lipolysis, they work synergically to increase de novo lipogenesis, which, in turn, might reduce the amounts of circulating FFA [65,66]. Further investigation of the interaction between GCs and insulin is extensive, and beyond the scope of this review.

Like GCs, growth hormone (GH) is known to have catabolic effects on WAT, inducing lipolysis [67]. However, the effects of GH on lipolysis can vary depending on the presence or absence of GCs. Incubation of omental AT depot from female subjects with DEX for 48 h led to increased *ATGL*, *LIPE*, and *PLIN1* (perilipin) mRNA levels. However, this increase in lipolytic enzymes’ mRNA did not translate to an increase in lipolysis, as measured by glycerol release. In addition, when tissues were incubated with GH for 48 h, only *LIPE* mRNA levels increased, with no increase in lipolysis. Only when the tissues were incubated with DEX and GH did a 39% increase in lipolysis occur, when compared to DEX alone. This indicates synergic effects between DEX and the GH. The increase in lipolysis was paired with a 47% and 41% increase in *LIPE* and *PLIN1* mRNA levels, respectively, compared to DEX alone, while *ATGL* mRNA level remained unchanged. These results suggest that DEX and GH are both required to alter the lipolytic rate, through a synergic mechanism [68]. GH works to increase lipolysis through activation of the β-adrenergic receptor, leading to an increase in *LIPE* mRNA levels, and activation of HSL via the PKC pathway, which can increase HSL phosphorylation [69]. In another study, however, cortisol and GH showed opposing effects on lipolysis in human SAT in the presence of insulin. Both male and female human SAT showed a decrease in lipolysis in response to cortisol, and an increase in lipolysis with the presence of GH [70]. These results were similar to those from in vivo experiments in humans, where cortisol and GH showed additive, lipolytic effects [71]. However, it is important to note that in the Ottosson study [70], insulin was also present in the culture medium. It shows that, when insulin and GH are both present, basal lipolytic rate might be increased, compared to when GH is alone [72]. It is possible that GCs and GH work through multiple pathways and may be dependent on species and type of tissue studied. In addition, presence, or absence, of insulin in the cultural medium might have an impact on the results of these studies, making the presence of insulin an important requirement in determining the effects of GH and GC on lipolysis [70].

## 5. GC Availability and 11-β Hydroxysteroid Dehydrogenase Type 1

A factor that can affect GC availability within AT is activity of 11-β hydroxysteroid dehydrogenase type 1 (11β-HSD1). As a prereceptor enzyme, 11β-HSD1 functions and converts the inactive GC to its active form, leading to increased GC availability within the cell [73,74]. Incubation of 3T3-L1 differentiated preadipocytes with CORT (0.01–0.1 µmol/L) for 24 h increased 11βHSD1, as well as glycerol release, in a dose-dependent manner [75]. However, the results of this study were contradictory to human AT studies, [76], in which the addition of 10 nM DEX to human omental AT explants for 48 h reduced 11β-HSD1 mRNA levels in the tissue. In male C57BL/6J mice, chronic exogenous CORT (7 weeks, 100 μg/mL in drinking water) led to higher body weight and FFA levels, compared to the control group [77]. This increase was parallel to a two- and 2.3-fold upsurge, respectively, in *11β-hsd1* mRNA and CORT levels, in extracted epididymal fat pad from mice. Following that, WAT *Atgl* and *Lipe* mRNA levels increased 3.0- and 3.5-fold, respectively, in CORT-treated mice, compared to vehicle-treated mice (1% ethanol was used as vehicle). In humans, in vivo administration of 4 mg of DEX per day for four days reduced *11β-hsd1* mRNA levels in the tissue [78]. This contradiction in results could be attributed to the varying types of GC exposure, or the subjects of the study (humans vs. mice). It is also possible that DEX decreased *11β-hsd1* mRNA levels, while CORT increased them. More detailed studies, with controlled levels of both DEX and CORT, would help determine the effects of GC exposure on 11β-HSD1 mRNA or protein levels.

The presence of corticosteroid-binding globulin (CBG) may also affect GC amounts in the body. CBG carries GCs in the blood, and high GC or insulin levels can inhibit CBG secretion [79]. CBG deficiency may decrease total circulating CBG-bound GC in the body, while increasing free circulating GC [80], and there are more observations of this effect in female rather than male mice [81]. As CBG may be an important in determining the availability of GCs in the body, we suggest further investigation into CBG as a tool to mitigate the negative side effects of GCs on lipid metabolism.

## 6. Loss of 11β-HSD1 Activity and Glucocorticoid Receptor in WAT

In addition to the studies in which GCs are introduced to the environment (gain of function studies), there are also loss of function genomic studies that focus on the lipolytic actions of GCs by eliminating the 11β-HSD1 or the GR.

Silencing of epididymal fat *11β-hsd1* using shRNA methods reduced CORT levels in both control and CORT-treated mice, and dramatically improved insulin sensitivity and body composition [77]. There was a parallel reduction in HSL and ATGL mRNA and protein levels. This shows that 11β-HSD1 has a regulatory effect on CORT availability in VAT, and its silence could alter the lipolytic rate. One mechanism of action by which CORT could affect lipolysis is through the AMPK pathway. AMPK is a negative regulator of HSL [82,83] and is reduced in the AT when GCs are added [77]. Silencing of *11β-hsd1* expression stops CORT-induced inhibition of pThr172 AMPK, which leads to the inhibition of HSL protein and reduced lipolysis [51]. The results of this study provide valuable information for basic pathways of GC function in AT, and AMPK silencing could be considered as a potential method to reduce negative side effects of GC treatment. However, in this study, *11β-hsd1* was silenced systemically and not in the AT alone.

Recent studies in which 11β-HSD1 is silenced locally (FKO, adipocyte-Cre crossed with floxed homozygous *11β-hsd1*) in the AT have found similar trends to those in systemic *11β-hsd1* KO mice. In this study, CORT-treated FKO mice were protected from the CORT-induced increase in SAT and WAT weight, increase in ATGL and HSL mRNA and protein levels, as well as increase in serum FFA, compared to control mice. FKO mice were also partially protected from CORT-induced Cushing’s syndrome [84]. Overall, these studies suggest that systemic or local inhibition of 11β-HSD1 in AT can be beneficial in overcoming some of the side effects of chronic GC exposure. However, this inhibition does not prevent GC-GR binding and, therefore, if GC is circulating, it can still bind to the GR and likely impact lipolysis via upregulation of the GC-sensitive AT lipases. We need long-term human trials to determine potential risks and side effects before 11β-HSD1 inhibition becomes a standard mode of therapy.

Elimination of GR can also act as a method for examining the effects of GC. Mice with a global GR knockout cannot survive long after birth due to immature lung development [85]. Tissue-specific GR knockout models, however, can be used to study the effects of GCs on various metabolic functions. A study using adipocyte-specific GR knockout (*Gr*^∆Adip^, adipoq-cre crossed with *Nr3c1* floxed mice) in mice found reduced lipolysis (as measured by NEFA release) from epididymal WAT compared to the WT [86]. In addition, these mice had reduced age- and diet-induced obesity, without affecting food intake. However, in a prolonged fasting state (48 h in mice), *Gr*^∆Adip^ mice had twice the body fat mass compared to the control group, along with enlarged WAT and increased adipocyte size, while simultaneously exhibiting lower plasma glycerol and NEFA. This reduction in lipolysis correlated with a reduction in ATGL mRNA and protein, while the HSL mRNA and protein levels remained the same. It is possible that the extreme stress of fasting for 48 h caused the β-adrenergic receptor to inhibit lipolytic pathways. Even though the *Gr*^∆Adip^ mice fed a high fat diet had a higher body fat mass after a prolonged fast than the control mice, the total body weight of both groups was similar. A normal body in a prolonged fasting stage will increase lipolysis to provide fuel, preserving muscle mass. In *Gr*^∆Adip^ mice, it may be that the absence of GRs reduces lipolysis, and muscle mass, of necessity, becomes a source of fuel. These results suggest that further work into adipocyte GR inhibition may yield options to influence age- and diet-induced obesity [86].

ATGL is regulated directly downstream of the GR activation, as evident from another AT-specific GR knockout (AGRKO, *adiponectin*-Cre mice crossed with the *Nr3c1* floxed mice) mouse model. These animals had significantly decreased ATGL protein and lipolysis levels (measured by non-esterified fatty acids (NEFA) and glycerol release) at both baseline and post-isoproterenol treatment, while HSL protein levels did not change. In addition, these animals were protected against DEX-induced metabolic dysfunction [87]. The results of this study were confirmed by a recent study that showed adipocyte GR knockout can be a possible model for overcoming the side effects of excess GCs [88]. A possible reason for why only one of ATGL or HSL is affected may be that ATGL protein expression is increased downstream of GR activation. Further studies may find a possible mechanism for changes in HSL mRNA or protein expression in GC exposure. It is also possible that GCs can affect HSL mRNA and protein expression in rats more than mice, as the studies that used primary rat epididymal adipocytes showed changes in HSL mRNA and protein level in response to GC. We also need more studies to find the applicability of adipocyte GR knockout in humans and its potential effects on adipose tissue function and lipid regulation, especially since the abovementioned GR knockout studies are primarily studied in WAT and do not account for direct GCs in BAT. More BAT-specific GR KO models can test whether the GC function in WAT is conserved in BAT.

Table 1 includes a summary of loss of function studies in WAT.

## 7. Effects of GCs on BAT Lipid Metabolism

BAT uses stored fatty acids as a source of heat production [32]. An increase in BAT thermogenic activity is one suggested therapeutic method to decrease AT lipid accumulation and prevent the development of obesity and other metabolic diseases [39]. GCs reduce the BAT-specific thermogenic and metabolic characteristics and induce whitening in BAT [89,90]. GCs also increase lipid accumulation in BAT, while decreasing non-shivering thermogenesis [40]. However, how GCs disrupt BAT thermogenic function remains largely unknown. Previous work suggests that these effects are not exerted directly through the GR in BAT [91].

A study performed in male rats showed that the addition of CORT (0.1–0.5 mg/mL) to drinking water for 21 days induced the remodelling of BAT to WAT, resulting in an increase in whole-body adiposity. Representative BAT samples from these rats documented a reduction in UCP1 mRNA and protein expression along with an increase in WAT-specific gene expression, such as fatty acid synthase, as responsible for lipid accumulation [89]. A study of diet-induced obese mice subjected to a seven week treatment of DEX (5 mg/kg) injection every other day showed a decrease in BAT *ucp1* mRNA expression and increased body fat percentage [92]. In other studies, *11β-hsd1^−/−^* models appear to be resistant to GC-induced reduction in BAT-specific genes, including *ucp1* [93,94].

Together, these studies indicate that limiting GC exposure improves BAT function or activity and lowers excess lipid accumulation. However, using an adipocyte-specific GR KO model, *Gr*^ΔAdip^ study showed that the lack of GR in adipocytes can reduce cold-induced thermogenic activity in BAT without affecting UCP1 mRNA and protein levels or BAT lipolysis [86]. This experiment suggested that the reduction in thermogenic activity of BAT is due to a reduction in external FA supply (i.e., from WAT), due to GC exposure, rather than the internal FA supply from BAT. Recent studies agree with the theory that cold-induced thermogenesis in BAT is dependent on external fuel supply [95,96,97].

Other recent work develops the idea that GCs can induce obesity independent of the UCP1. For example, GC-induced obesity develops to the same extent in WT and UCP1 KO models [98]. In addition, one recent study by Luijten et al. showed that GCs only decrease relative UCP1 protein compared to total tissue protein, and not the total UCP1 protein available in the cell tissue [99]. We can conclude that GCs do not alter the true amount of UCP1 protein available in the tissue, and their obesogenic effects on the body are independent of BAT UCP1.

All the abovementioned studies about the effects of GCs on BAT were done in rodents. In human studies, DEX stimulates proliferation, differentiation, and function of human BAT cells [100]. Additionally, acute (36 h) GC exposure in male human subjects increases UCP1 protein levels and BAT thermogenic activity, while chronic GC exposure (for at least two weeks) decreases BAT activation [101]. It is possible that there are inter-species differences between humans and rodents in terms of BAT activation and function; more study can determine if this is true.

**Figure 2:**
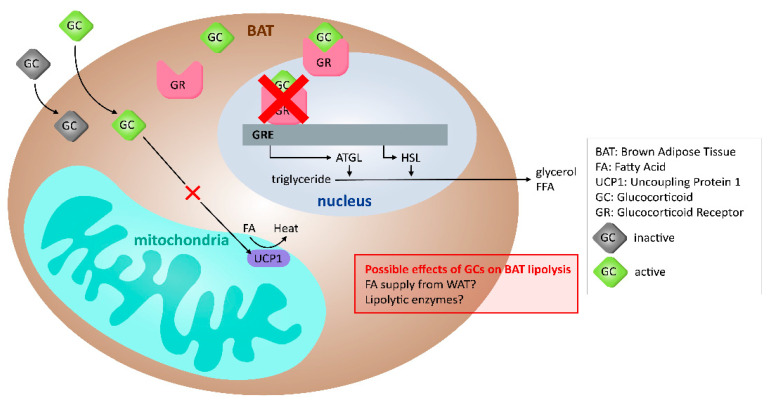
Effects of GCs on lipid metabolism in BAT. The effects of GCs on lipolysis in BAT appear to be independent of UCP1 and the GR. Multiple potential mechanisms in BAT include the effects of the extracellular FA availability on GCs’ induced lipid accumulation.

The pathways underlying the actions of GCs on BAT, and possible role in lipolysis, are largely unknown. There is no clear conclusion on the current data about how, and if, GCs deplete BAT of its thermogenic activity, and what role the GR plays in BAT lipid accumulation. It is possible that the genomic effects that were observed downstream of GC activation in WAT are also conserved in BAT. In addition, the effects of GCs on altering BAT thermogenic activity could be due to an alteration in the utilization of FA from external sources (such as WAT) instead of a true effect on BAT itself. Studies focusing on the effects of GCs on BAT and its associated organs, with an emphasis on understanding the possible inter-species differences in BAT, can yield more information to determine GC effects on BAT lipolysis. A summary of the effects of GCs on lipolysis in BAT is shown in Figure 2 and Table 2.

## 8. Non-Genomic Effects of GCs on Lipolysis in AT

There is less study on the non-genomic effects of GCs on lipolysis than on the genomic effects in AT. The non-genomic mechanisms likely occur within minutes after GC exposure and may or may not be dependent on the cytosolic or membrane-bound GR [24]. In theory, these non-genomic actions would disappear rapidly with the removal of the hormone from circulation, while the genomic actions may be sustained. If an action is deemed dependent on the GR, it must, therefore, be insensitive to the gene transcription inhibition in order to be considered non-genomic [23]. This provides additional complications to how GCs are studied. In this section, we consider changes to enzyme activity levels and phosphorylation states (i.e. cAMP and PKA) to be considered non-genomic actions of GCs.

As with the genomic actions of GCs, non-genomic effects can vary vastly depending on tissue specificity. Studies of GC exposure showed rapidly reduced intracellular [Ca^2+^] levels in human bronchial epithelial cells [102], rat thymocytes [103], and mouse neuroblastoma [104] but increased intracellular [Ca2+] in rat vascular smooth muscle cells [105]. In breast cancer cells, cortisol exposure led to rapid induction of DNA damage, due to increased levels of reactive oxygen and reactive nitrogen species [106].

Differentiated mouse 3T3-L1 preadipocytes were incubated with 1–10 uM of CORT for 48 h increased lipolysis, while glycerol release decreased with concentrations beyond 10 uM [47]. The presence of CORT led to a decrease in cAMP levels, independent of its concentration. The lipolytic effects normalized once the CORT was removed, suggesting that some immediate, non-genomic functions might be involved. Concluding that, while the lipolytic effects of CORT in lower concentrations are mediated by increasing expression of ATGL protein, the antilipolytic effects in higher CORT concentrations are mediated through a non-genomic action via the cAMP pathways.

Another body of work focuses on the actions of GCs through the PIK3R1 pathway in adipocytes [38]. PIK3R1, a GR target gene in 3T3-L1 and visceral adipocytes, is linked to a reduction in insulin sensitivity. Since insulin is linked to the suppression of lipolysis through the PI3K-AKT-PDE3B-dependent pathway and reduction in cAMP [107,108,109], this GC-induced enhancement in PIK3R1 could lead to the induction of lipolysis. AT-specific *Pik3r1* null mice (AKO) showed that, after the addition of DEX for 24 h, ATGL protein and mRNA expression increased in both WT and AKO mice. This increase in ATGL protein levels led to an increase in lipolysis in WT, but not in the AKO mice [38]. The lipid droplet PKA activity levels failed to increase after the addition of DEX in AKO mice, while PKA activity increased in WT mice. AKO mice had reduced phosphorylation of perilipin, which is required for recruitment of phosphorylated HSL into the lipid droplet [110,111,112]. Therefore, the reduction in *Pik3r1* gene in epididymal and inguinal AT led to impairment in GC-induced lipolysis through failure in the PKA-mediated translocation of HSL into the lipid droplet, suggesting non-genomic effects of GCs. However, the pathway involved in GC-induced PKA increase in the lipid droplet is not clear. Moreover, the involvement of PI3K in GC-induced pathways has also been suggested in other tissues. For example, in human endothelial cells, GCs rapidly activated PI3K and its downstream target genes, leading to activation of the nitric oxide pathway required for vasorelaxation after ischemic injury [113]. We need more investigation to understand how GCs regulate PKA-mediated pathways through non-genomic mechanisms.

**Figure 3:**
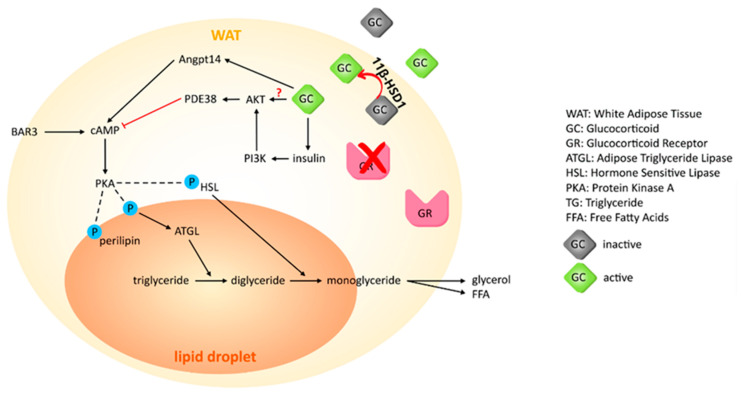
Non-genomic effects of GCs on lipolysis in WAT. The non-genomic effects are independent of ATGL/HSL gene or protein expression. Multiple suggested non-genomic mechanisms include AKT-PDE3B pathway, the ANGPTL4-cAMP pathway, and the insulin-PI3K pathway. The ANGPTL4 is a GR target gene that may be classified as inducing possible non-genomic mechanisms through its activation in cAMP. The non-genomic pathways affect lipolysis by regulating activity of HSL/perilipin through affecting phosphorylation. 11β-HSD1: 11-β hydroxysteroid dehydrogenase type, AKT: Protein Kinase B, PDE3B: phosphodiesterase 3B, ANGPTL4: Angiopoietin-like protein 4, cAMP: Cyclic adenosine monophosphate, PI3K: Phosphatidylinositol 3-kinase, BA3R: Beta-3 adrenergic receptor.

Possible genomic and non-genomic actions of GC-induced lipolysis in adipocytes may also involve the ANGPTL4 protein, another GR target gene, which acts upstream of PIK3R1, and directly increases cAMP levels, inducing lipolysis. ANGPTL4 is transcriptionally upregulated by GR binding, however, it is important for increasing cAMP levels and the phosphorylation of downstream lipolytic enzymes. One study with *Angptl4*-null mice had a significant reduction in DEX-induced phosphorylation of HSL and PLIN1 initiated by PKA [114], suggesting that ANGPTL4 works upstream of PIK3R1, which is dependent on ANGPTL4. At the same time, it is not clear whether ANGPTL4 is dependent on PIK3R1 to induce these effects. ANGPTL4 is also found in BAT and has a catabolic role in triglyceride metabolism [115], but we do not know whether it has a role in the non-genomic effects in BAT. With no published work on non-genomic mechanisms of GCs on human differentiated adipocytes, there is an opportunity to gain much valuable knowledge with additional research. The non-genomic effects of GCs on lipolysis in WAT are shown in Figure 3.

To further complicate a complex system, genomic and non-genomic actions can also happen simultaneously. For example, Xu et al. [57] found increases in lipolysis through genomic effects in rat primary epididymal adipocytes, while also observing the use of non-genomic pathways. They showed an increase in cAMP and PKA after 24 h of DEX exposure. However, using a PKA inhibitor (H89) led to decreased PKA activity, as well as reduced FFA and glycerol release. This suggests that some of the released FFA and glycerol in the study could be attributed to the effects of PKA, as opposed to a direct result of *Atgl* and *Lipe* mRNA transcription. These results agree with the results from the AKO mice study, both suggesting that the PKA activation pathway could be an important factor in non-genomic mechanisms of lipolysis in AT. Table 3 summarizes the genomic and non-genomic actions of GCs on lipolysis in AT.

GCs can also induce preadipocyte differentiation in 3T3-L1 cells through non-genomic actions, as seen when 3T3-L1 cells were incubated with 250 nM of DEX for 48 h [116]. Possible mechanisms may have resulted from the interaction of the GR with associated proteins, such as the CCAAT/enhancer binding protein (C/EBP)β, and enhancement of its transcriptional activity. C/EBPβ is a key transcription factor that induces the expression of major adipocyte differentiation regulators, such as PPARγ [117]. Distinguishing between adipogenicity and lipolytic actions of DEX requires the dose and timeframe of exposure. Even though DEX led to preadipocyte differentiation in this study, the amount of the DEX (250 nM) was beyond normal physiological levels, whereas lower DEX dose studies showed lipolytic effects [55]. Likewise, timeframe (48 h vs. 6 days) has an important influence; preadipocytes were present in different differentiation stages in each of these studies. It is also shown that chronic CORT exposure (48 h) in non-differentiated 3T3-L1 cells and male Sprague–Dawley rats can increase adipogenesis by enhancing preadipocyte differentiation [47].

GCs can affect adipocytes differently based on the differentiation status of cells. For example, in preadipocytes, GC-GR binding can lead to the activation of heart and neural crest derivatives expressed 2 (HAND2), a transcription factor necessary for early adipocyte differentiation [118]. Silencing *HAND2* prevents commitment to the adipose lineage in human stem cells and downregulates lipid metabolism genes, such as *HSL*. In early adipocytes, *HAND2* silencing can prevent formation of lipid droplets, preventing mature adipocytes from forming. However, its silencing has no consequences in lipid metabolism in mature adipocytes, indicating that the differentiation stage of the adipocytes dictates the effects of GCs on lipolysis. It is possible that GC exposure leads to the recruitment of non-genomic pathways in certain doses and timeframes, while recruiting genomic actions in other doses and time frames. More studies are needed to determine critical points of differentiation and controlled time-dependent experiments, to find the dose-dependent effect of GCs on lipid metabolism in all varying types of ATs. In addition, the genomic actions of GCs and their effects on BAT thermogenic activity and impact on UCP1 protein expression have been largely studied, while the non-genomic effects have not.

In summary, the majority of the studies we reviewed conclude that the addition of DEX and CORT provide varying results in the regulation of lipolytic enzymes in WAT. The addition of DEX alone can alter ATGL and lipolytic rate, with no significant effect on HSL protein levels, while the addition of CORT alone can alter the levels of both ATGL and HSL proteins. Only when DEX is paired with other substances, such as pioglitazone, can it affect both ATGL and HSL levels. In addition, while DEX treatment in WAT decreased *11β-hsd1* mRNA levels, CORT exposure increased them. Therefore, we conclude that treatment, dose, and time exposure are very important factors in determining the exact effects of GCs in lipid metabolism in AT. Some of the lipolytic effects of GCs in most of these studies can be attributed to the non-genomic pathways, mainly through affecting phosphorylation sites in HSL downstream of PKA. It is possible that genomic actions can affect ATGL mRNA and protein level, and non-genomic actions can influence the phosphorylation and function of HSL.

The genomic and non-genomic effects of GCs on lipolysis in BAT are mainly unknown and controversial. Some studies suggest that GC can diminish lipolysis in BAT through reduction in UCP1, while others suggest that this reduction in lipolysis is independent of UCP1, and dependent on external fuel source. It is crucial for further studies to determine the underlying mechanism for the non-genomic actions of GCs in lipid metabolism in the WAT and BAT. A clear understanding of the basic physiology of the AT and GC pathways and the effects of high levels of endogenous and prescribed GCs will be invaluable in the effective management of chronic diseases such as obesity, diabetes, and cancer.

## Figures and Tables

**Table 1:** Summary of loss of GC function studies on lipolysis in WAT.

Molecule Inhibited	Effects of Lipolysis	Effects on ATGL	Effects on HSL	Effects on Other Molecules
11β-HSD1 in epididymal fat of mice [77] (shRNA injection)	Decrease	Decrease	Decrease	Decreased CORT in CORT-treated mice compared to WT
11β-HSD1 in AT [84] (Adipocyte-Cre crossed with floxed *11β-hsd1*, C57BL/6J mice)	Decrease compared to CORT-treated WT mice	Decrease	Decrease	
Epididymal adipocyte GR KO in mice [86] (Adipoq-Cre crossed with *Nr3c1* floxed, C57BL/6J mice)	Decrease	Decrease	None	
Adipocyte GR KO in mice [87] Adipoq-Cre crossed with *Nr3c1* floxed, C57BL/6J mice	Decrease in isoproterenol-stimulated glycerol release	Decrease	None	

**Table 2:** Summary of effects of GC gain/loss of function of GCs in BAT.

Cell/Tissue/Species	Type of Manipulation	GC Concentration	Duration of Exposure	Effects on UCP1	Effects on Lipolysis	Effects on Thermogenesis
Male Wistar rat [89]	CORT	0.1–0.5 mg/mL	21 days	Decrease	Decrease	N/A
Male C57BL/6J Mice [90]	CORT	5 mg/kg	1 week	Decrease	Decrease	N/A
Male Mice [86] (Adipoq-Cre crossed with *Nr3c1* floxed C57BL/6 mice)	Adipocyte-specific GR KO	N/A	N/A	None	None	Decrease
Male C57BL/6J Mice [98]	UCP1 KO	50 µg/mL drinking water	2 weeks	KO	Not changed compared to CORT-treated WT	Not changed compared to CORT-treated WT
Male mice [91] (*Ucp1-*Cre^ERT2^ crossed with *Nr3c1* floxed C57BL/6 mice)	BAT-specific GR KO induced by tamoxifen	0.1 mg/kg injected subcutaneously	150 min	None	None	None
Human BAT depot [101]	Cortisol	0–1000 nM	24 h	Increase (peak increase at 100 nM of Cortisol)	N/A	Increase (peak increase at 100 nM)
Male human [101]	prednisolone	10 mg every 12 h	36 h	Increase	Increase (NEFAs)	Increase
Male human patients [101], retrospective study	oral GC therapy	unknown	>2 weeks	Decrease	N/A	Decrease

**Table 3:** Summary of gain of GC function studies on lipolysis in WAT.

Cells/Tissue/Species	Stimulation	Amount of GC	Duration of GC Exposure	Effect on Lipolysis	Effect on ATGL	Effect on HSL	Effect on Other Molecules	Genomic/Non-genomic
Differentiated 3T3-L1 cells [55]	DEX	20 nM	6 days	Increase	Increase	None		Gen
Rat primary epididymal adipocytes [56]	DEX	100–1000 nM	24 h	Increase	N/A	Increase		Gen
Rat primary epididymal adipocytes [56]	DEX	1000 nM	4 h	None	N/A	None		Gen
Rat primary epididymal adipocytes [57]	DEX	0–1000	24 h	Increase (peak at 1000 nM)	Increase	Increase		Gen
Female human omental depot [68]	DEX	50 nM	48 h	None	Increase	Increase		Gen
Male C57BL/6J Mice [58]	DEX	1 mg/kg/d	12 weeks	Increase	Increase	None		Gen
Differentiated 3T3-L1 cells [55]	DEX + pioglitazone	10–100 nM	6 days	Increase compared to basal Decrease compared to DEX alone	Increase	Increase	Increase in GK and PEPK (lipogenic enzymes)	Gen
Female human omental depot [68]	DEX + GH	50 nM	48 h	Increase	Increase	Increase		Gen
Subcutaneous abdominal depot [70]	Cortisol	1000 nmol/L	3 days	Decrease	N/A	N/A		Gen
Subcutaneous abdominal depot [70]	Cortisol + GH	1000 nmol/L	3 days	Increase compared to cortisol alone	N/A	N/A		Gen
Human male [71]	Cortisol	2 μg/kg/min	3 h	Increase	N/A	N/A		Gen
Human male [71]	Cortisol + GH	2 μg/kg/min	3 h	Increase compared to placebo AND Cortisol alobe	N/A	N/A		Gen
AT–specific Pik3r1-null mice (Adipoq-Cre cross with floxed *Pik3r1*) [38]	DEX	10 mg/kg body	24 h	None compared to placebo, Decreased compared to DEX-treated WT	Increase compared to placebo	Increased non-significantly compared to WT	Decrease in lipid droplet PKA	Gen and Non-Gen
Angptl4-null mice (Mixed background, C57BL6:129 Sv [114]	DEX	(5 mg/kg body	24 h	Decrease compared to WT	Decrease	None	Decrease in PKA-inducedphosphorylation of HSL	Gen and Non-Gen
Rat primary epididymal adipocytes [57]	DEX + H89	0–1000 nM	24 h	Decrease compared to DEX alone	N/A	N/A	Decrease in PKA	Non-Gen

## References

[1] Miller W.L., Auchus R.J. (2011). The molecular biology, biochemistry, and physiology of human steroidogenesis and its disorders. Endocr. Rev..

[2] Rhen T., Cidlowski J.A. (2005). Antiinflammatory action of glucocorticoids--new mechanisms for old drugs. N. Engl. J. Med..

[3] Group R.C., Horby P., Lim W.S., Emberson J.R., Mafham M., Bell J.L., Linsell L., Staplin N., Brightling C., Ustianowski A. (2021). Dexamethasone in Hospitalized Patients with Covid-19. N. Engl. J. Med..

[4] Whirledge S., DeFranco D.B. (2018). Glucocorticoid Signaling in Health and Disease: Insights From Tissue-Specific GR Knockout Mice. Endocrinology.

[5] Busada J.T., Cidlowski J.A. (2017). Mechanisms of Glucocorticoid Action During Development. Curr. Top. Dev. Biol..

[6] Holmes M.C., Abrahamsen C.T., French K.L., Paterson J.M., Mullins J.J., Seckl J.R. (2006). The mother or the fetus? 11beta-hydroxysteroid dehydrogenase type 2 null mice provide evidence for direct fetal programming of behavior by endogenous glucocorticoids. J. Neurosci..

[7] Wyrwoll C.S., Seckl J.R., Holmes M.C. (2009). Altered placental function of 11beta-hydroxysteroid dehydrogenase 2 knockout mice. Endocrinology.

[8] Beijers R., Jansen J., Riksen-Walraven M., de Weerth C. (2010). Maternal prenatal anxiety and stress predict infant illnesses and health complaints. Pediatrics.

[9] Madalena K.M., Lerch J.K. (2017). The Effect of Glucocorticoid and Glucocorticoid Receptor Interactions on Brain, Spinal Cord, and Glial Cell Plasticity. Neural Plast..

[10] Dubovsky A.N., Arvikar S., Stern T.A., Axelrod L. (2012). The neuropsychiatric complications of glucocorticoid use: Steroid psychosis revisited. Psychosomatics.

[11] Sandi C., Merino J.J., Cordero M.I., Touyarot K., Venero C. (2001). Effects of chronic stress on contextual fear conditioning and the hippocampal expression of the neural cell adhesion molecule, its polysialylation, and L1. Neuroscience.

[12] Dallman M.F., la Fleur S.E., Pecoraro N.C., Gomez F., Houshyar H., Akana S.F. (2004). Minireview: Glucocorticoids--food intake, abdominal obesity, and wealthy nations in 2004. Endocrinology.

[13] Weikum E.R., Knuesel M.T., Ortlund E.A., Yamamoto K.R. (2017). Glucocorticoid receptor control of transcription: Precision and plasticity via allostery. Nat. Rev. Mol. Cell Biol..

[14] Yu C.Y., Mayba O., Lee J.V., Tran J., Harris C., Speed T.P., Wang J.C. (2010). Genome-wide analysis of glucocorticoid receptor binding regions in adipocytes reveal gene network involved in triglyceride homeostasis. PLoS ONE.

[15] Arriza J.L., Weinberger C., Cerelli G., Glaser T.M., Handelin B.L., Housman D.E., Evans R.M. (1987). Cloning of human mineralocorticoid receptor complementary DNA: Structural and functional kinship with the glucocorticoid receptor. Science.

[16] Urbanet R., Nguyen Dinh Cat A., Feraco A., Venteclef N., El Mogrhabi S., Sierra-Ramos C., Alvarez de la Rosa D., Adler G.K., Quilliot D., Rossignol P. (2015). Adipocyte Mineralocorticoid Receptor Activation Leads to Metabolic Syndrome and Induction of Prostaglandin D2 Synthase. Hypertension.

[17] Lefranc C., Friederich-Persson M., Foufelle F., Nguyen Dinh Cat A., Jaisser F. (2021). Adipocyte-Mineralocorticoid Receptor Alters Mitochondrial Quality Control Leading to Mitochondrial Dysfunction and Senescence of Visceral Adipose Tissue. Int. J. Mol. Sci..

[18] Ferguson D., Hutson I., Tycksen E., Pietka T.A., Bauerle K., Harris C.A. (2020). Role of Mineralocorticoid Receptor in Adipogenesis and Obesity in Male Mice. Endocrinology.

[19] Le Magueresse-Battistoni B. (2020). Adipose Tissue and Endocrine-Disrupting Chemicals: Does Sex Matter?. Int. J. Environ. Res. Public Health.

[20] Edwards C.R., Stewart P.M., Burt D., Brett L., McIntyre M.A., Sutanto W.S., de Kloet E.R., Monder C. (1988). Localisation of 11 beta-hydroxysteroid dehydrogenase--tissue specific protector of the mineralocorticoid receptor. Lancet.

[21] Rebuffat A.G., Tam S., Nawrocki A.R., Baker M.E., Frey B.M., Frey F.J., Odermatt A. (2004). The 11-ketosteroid 11-ketodexamethasone is a glucocorticoid receptor agonist. Mol. Cell Endocrinol..

[22] Viengchareun S., Le Menuet D., Martinerie L., Munier M., Pascual-Le Tallec L., Lombes M. (2007). The mineralocorticoid receptor: Insights into its molecular and (patho)physiological biology. Nucl. Recept Signal..

[23] Haller J., Mikics E., Makara G.B. (2008). The effects of non-genomic glucocorticoid mechanisms on bodily functions and the central neural system. A critical evaluation of findings. Front. Neuroendocrinol..

[24] Panettieri R.A., Schaafsma D., Amrani Y., Koziol-White C., Ostrom R., Tliba O. (2019). Non-genomic Effects of Glucocorticoids: An Updated View. Trends Pharmacol. Sci..

[25] Johnstone W.M., Honeycutt J.L., Deck C.A., Borski R.J. (2019). Nongenomic glucocorticoid effects and their mechanisms of action in vertebrates. Int. Rev. Cell Mol. Biol..

[26] Kusminski C.M., Bickel P.E., Scherer P.E. (2016). Targeting adipose tissue in the treatment of obesity-associated diabetes. Nat. Rev. Drug Discov..

[27] Coutinho A.E., Chapman K.E. (2011). The anti-inflammatory and immunosuppressive effects of glucocorticoids, recent developments and mechanistic insights. Mol. Cell Endocrinol..

[28] Clore J.N., Thurby-Hay L. (2009). Glucocorticoid-induced hyperglycemia. Endocr. Pract..

[29] Lee M.J., Pramyothin P., Karastergiou K., Fried S.K. (2014). Deconstructing the roles of glucocorticoids in adipose tissue biology and the development of central obesity. Biochim. Biophys. Acta..

[30] Dallman M.F., Pecoraro N.C., La Fleur S.E. (2005). Chronic stress and comfort foods: Self-medication and abdominal obesity. Brain Behav. Immun..

[31] Nieman L.K. (2015). Cushing’s syndrome: Update on signs, symptoms and biochemical screening. Eur. J. Endocrinol..

[32] Saely C.H., Geiger K., Drexel H. (2012). Brown versus white adipose tissue: A mini-review. Gerontology.

[33] Fox C.S., Massaro J.M., Hoffmann U., Pou K.M., Maurovich-Horvat P., Liu C.Y., Vasan R.S., Murabito J.M., Meigs J.B., Cupples L.A. (2007). Abdominal visceral and subcutaneous adipose tissue compartments: Association with metabolic risk factors in the Framingham Heart Study. Circulation.

[34] Oh T.H., Byeon J.S., Myung S.J., Yang S.K., Choi K.S., Chung J.W., Kim B., Lee D., Byun J.H., Jang S.J. (2008). Visceral obesity as a risk factor for colorectal neoplasm. J. Gastroenterol. Hepatol..

[35] Schapira D.V., Clark R.A., Wolff P.A., Jarrett A.R., Kumar N.B., Aziz N.M. (1994). Visceral obesity and breast cancer risk. Cancer.

[36] Lafontan M. (2008). Advances in adipose tissue metabolism. Int. J. Obes. (Lond.).

[37] Peckett A.J., Wright D.C., Riddell M.C. (2011). The effects of glucocorticoids on adipose tissue lipid metabolism. Metabolism.

[38] Kuo T., Chen T.C., Lee R.A., Nguyen N.H.T., Broughton A.E., Zhang D., Wang J.C. (2017). Pik3r1 Is Required for Glucocorticoid-Induced Perilipin 1 Phosphorylation in Lipid Droplet for Adipocyte Lipolysis. Diabetes.

[39] Becher T., Palanisamy S., Kramer D.J., Eljalby M., Marx S.J., Wibmer A.G., Butler S.D., Jiang C.S., Vaughan R., Schoder H. (2021). Brown adipose tissue is associated with cardiometabolic health. Nat. Med..

[40] Strack A.M., Bradbury M.J., Dallman M.F. (1995). Corticosterone decreases nonshivering thermogenesis and increases lipid storage in brown adipose tissue. Am. J. Physiol..

[41] Jeffery E., Church C.D., Holtrup B., Colman L., Rodeheffer M.S. (2015). Rapid depot-specific activation of adipocyte precursor cells at the onset of obesity. Nat. Cell Biol..

[42] Altshuler-Keylin S., Shinoda K., Hasegawa Y., Ikeda K., Hong H., Kang Q., Yang Y., Perera R.M., Debnath J., Kajimura S. (2016). Beige Adipocyte Maintenance Is Regulated by Autophagy-Induced Mitochondrial Clearance. Cell Metab..

[43] Chouchani E.T., Kajimura S. (2019). Metabolic adaptation and maladaptation in adipose tissue. Nat. Metab..

[44] Djurhuus C.B., Gravholt C.H., Nielsen S., Mengel A., Christiansen J.S., Schmitz O.E., Moller N. (2002). Effects of cortisol on lipolysis and regional interstitial glycerol levels in humans. Am. J. Physiol. Endocrinol. Metab..

[45] Divertie G.D., Jensen M.D., Miles J.M. (1991). Stimulation of lipolysis in humans by physiological hypercortisolemia. Diabetes.

[46] Roberge C., Carpentier A.C., Langlois M.-F., Baillargeon J.-P., Ardilouze J.-L., Maheux P., Gallo-Payet N. (2007). Adrenocortical dysregulation as a major player in insulin resistance and onset of obesity. Am. J. Physiol. Endocrinol. Metab..

[47] Campbell J.E., Peckett A.J., D’Souza A.M., Hawke T.J., Riddell M.C. (2011). Adipogenic and lipolytic effects of chronic glucocorticoid exposure. Am. J. Physiol. Cell Physiol..

[48] Green H., Meuth M. (1974). An established pre-adipose cell line and its differentiation in culture. Cell.

[49] Ruiz-Ojeda F.J., Ruperez A.I., Gomez-Llorente C., Gil A., Aguilera C.M. (2016). Cell Models and Their Application for Studying Adipogenic Differentiation in Relation to Obesity: A Review. Int. J. Mol. Sci..

[50] Gregoire F.M., Smas C.M., Sul H.S. (1998). Understanding adipocyte differentiation. Physiol. Rev..

[51] Moseti D., Regassa A., Kim W.K. (2016). Molecular Regulation of Adipogenesis and Potential Anti-Adipogenic Bioactive Molecules. Int. J. Mol. Sci..

[52] Bazhan N., Jakovleva T., Feofanova N., Denisova E., Dubinina A., Sitnikova N., Makarova E. (2019). Sex Differences in Liver, Adipose Tissue, and Muscle Transcriptional Response to Fasting and Refeeding in Mice. Cells.

[53] Benz V., Bloch M., Wardat S., Bohm C., Maurer L., Mahmoodzadeh S., Wiedmer P., Spranger J., Foryst-Ludwig A., Kintscher U. (2012). Sexual dimorphic regulation of body weight dynamics and adipose tissue lipolysis. PLoS ONE.

[54] Stahn C., Buttgereit F. (2008). Genomic and nongenomic effects of glucocorticoids. Nat. Clin. Pract. Rheumatol..

[55] Hasan A.U., Ohmori K., Hashimoto T., Kamitori K., Yamaguchi F., Rahman A., Tokuda M., Kobori H. (2018). PPARgamma activation mitigates glucocorticoid receptor-induced excessive lipolysis in adipocytes via homeostatic crosstalk. J. Cell. Biochem..

[56] Slavin B.G., Ong J.M., Kern P.A. (1994). Hormonal regulation of hormone-sensitive lipase activity and mRNA levels in isolated rat adipocytes. J. Lipid Res..

[57] Xu C., He J., Jiang H., Zu L., Zhai W., Pu S., Xu G. (2009). Direct effect of glucocorticoids on lipolysis in adipocytes. Mol. Endocrinol..

[58] Harvey I., Stephenson E.J., Redd J.R., Tran Q.T., Hochberg I., Qi N., Bridges D. (2018). Glucocorticoid-Induced Metabolic Disturbances Are Exacerbated in Obese Male Mice. Endocrinology.

[59] Yilmaz-Aydogan H., Kurnaz O., Kurt O., Akadam-Teker B., Kucukhuseyin O., Tekeli A., Isbir T. (2011). Effects of the PPARG P12A and C161T gene variants on serum lipids in coronary heart disease patients with and without Type 2 diabetes. Mol. Cell Biochem..

[60] Fong W.H., Tsai H.D., Chen Y.C., Wu J.S., Lin T.N. (2010). Anti-apoptotic actions of PPAR-gamma against ischemic stroke. Mol. Neurobiol..

[61] Richelsen B., Pedersen S.B., Moller-Pedersen T., Bak J.F. (1991). Regional differences in triglyceride breakdown in human adipose tissue: Effects of catecholamines, insulin, and prostaglandin E2. Metabolism.

[62] Lonnroth P., Smith U. (1986). The antilipolytic effect of insulin in human adipocytes requires activation of the phosphodiesterase. Biochem. Biophys. Res. Commun..

[63] Stimson R.H., Anderson A.J., Ramage L.E., Macfarlane D.P., de Beaux A.C., Mole D.J., Andrew R., Walker B.R. (2017). Acute physiological effects of glucocorticoids on fuel metabolism in humans are permissive but not direct. Diabetes Obes. Metab..

[64] Dallman M.F., Strack A.M., Akana S.F., Bradbury M.J., Hanson E.S., Scribner K.A., Smith M. (1993). Feast and famine: Critical role of glucocorticoids with insulin in daily energy flow. Front. Neuroendocrinol..

[65] Gathercole L.L., Morgan S.A., Bujalska I.J., Hauton D., Stewart P.M., Tomlinson J.W. (2011). Regulation of lipogenesis by glucocorticoids and insulin in human adipose tissue. PLoS ONE.

[66] Wang Y., Jones Voy B., Urs S., Kim S., Soltani-Bejnood M., Quigley N., Heo Y.R., Standridge M., Andersen B., Dhar M. (2004). The human fatty acid synthase gene and de novo lipogenesis are coordinately regulated in human adipose tissue. J. Nutr..

[67] Moller N., Jorgensen J.O. (2009). Effects of growth hormone on glucose, lipid, and protein metabolism in human subjects. Endocr. Rev..

[68] Fain J.N., Cheema P., Tichansky D.S., Madan A.K. (2008). Stimulation of human omental adipose tissue lipolysis by growth hormone plus dexamethasone. Mol. Cell Endocrinol..

[69] Kopchick J.J., Berryman D.E., Puri V., Lee K.Y., Jorgensen J.O.L. (2020). The effects of growth hormone on adipose tissue: Old observations, new mechanisms. Nat. Rev. Endocrinol..

[70] Ottosson M., Lonnroth P., Bjorntorp P., Eden S. (2000). Effects of cortisol and growth hormone on lipolysis in human adipose tissue. J. Clin. Endocrinol. Metab..

[71] Djurhuus C.B., Gravholt C.H., Nielsen S., Pedersen S.B., Moller N., Schmitz O. (2004). Additive effects of cortisol and growth hormone on regional and systemic lipolysis in humans. Am. J. Physiol. Endocrinol. Metab..

[72] Kang E.S., Betts D., Fain J.N., Bahouth S.W., Myers L.K. (1993). Chronic exposure of rat fat cells to insulin enhances lipolysis and activation of partially purified hormone-sensitive lipase. Diabetes.

[73] White P.C., Mune T., Agarwal A.K. (1997). 11 beta-Hydroxysteroid dehydrogenase and the syndrome of apparent mineralocorticoid excess. Endocr. Rev..

[74] Masuzaki H., Paterson J., Shinyama H., Morton N.M., Mullins J.J., Seckl J.R., Flier J.S. (2001). A transgenic model of visceral obesity and the metabolic syndrome. Science.

[75] Campbell J.E., Fediuc S., Hawke T.J., Riddell M.C. (2009). Endurance exercise training increases adipose tissue glucocorticoid exposure: Adaptations that facilitate lipolysis. Metabolism.

[76] Fain J.N., Cheema P., Madan A.K., Tichansky D.S. (2010). Dexamethasone and the inflammatory response in explants of human omental adipose tissue. Mol. Cell Endocrinol..

[77] Wang Y., Yan C., Liu L., Wang W., Du H., Fan W., Lutfy K., Jiang M., Friedman T.C., Liu Y. (2015). 11beta-Hydroxysteroid dehydrogenase type 1 shRNA ameliorates glucocorticoid-induced insulin resistance and lipolysis in mouse abdominal adipose tissue. Am. J. Physiol. Endocrinol. Metab..

[78] Viguerie N., Picard F., Hul G., Roussel B., Barbe P., Iacovoni J.S., Valle C., Langin D., Saris W.H. (2012). Multiple effects of a short-term dexamethasone treatment in human skeletal muscle and adipose tissue. Physiol. Genomics.

[79] Fernandez-Real J.M., Grasa M., Casamitjana R., Pugeat M., Barret C., Ricart W. (1999). Plasma total and glycosylated corticosteroid-binding globulin levels are associated with insulin secretion. J. Clin. Endocrinol. Metab..

[80] Petersen H.H., Andreassen T.K., Breiderhoff T., Brasen J.H., Schulz H., Gross V., Grone H.J., Nykjaer A., Willnow T.E. (2006). Hyporesponsiveness to glucocorticoids in mice genetically deficient for the corticosteroid binding globulin. Mol. Cell Biol..

[81] Gulfo J., Castel R., Ledda A., Romero M.D.M., Esteve M., Grasa M. (2019). Corticosteroid-Binding Globulin is expressed in the adrenal gland and its absence impairs corticosterone synthesis and secretion in a sex-dependent manner. Sci. Rep..

[82] Greenberg A.S., Shen W.J., Muliro K., Patel S., Souza S.C., Roth R.A., Kraemer F.B. (2001). Stimulation of lipolysis and hormone-sensitive lipase via the extracellular signal-regulated kinase pathway. J. Biol. Chem..

[83] Londos C., Brasaemle D.L., Schultz C.J., Adler-Wailes D.C., Levin D.M., Kimmel A.R., Rondinone C.M. (1999). On the control of lipolysis in adipocytes. Ann. N. Y. Acad. Sci..

[84] Morgan S.A., McCabe E.L., Gathercole L.L., Hassan-Smith Z.K., Larner D.P., Bujalska I.J., Stewart P.M., Tomlinson J.W., Lavery G.G. (2014). 11beta-HSD1 is the major regulator of the tissue-specific effects of circulating glucocorticoid excess. Proc. Natl. Acad. Sci. USA.

[85] Cole T.J., Blendy J.A., Monaghan A.P., Krieglstein K., Schmid W., Aguzzi A., Fantuzzi G., Hummler E., Unsicker K., Schutz G. (1995). Targeted disruption of the glucocorticoid receptor gene blocks adrenergic chromaffin cell development and severely retards lung maturation. Genes. Dev..

[86] Mueller K.M., Hartmann K., Kaltenecker D., Vettorazzi S., Bauer M., Mauser L., Amann S., Jall S., Fischer K., Esterbauer H. (2017). Adipocyte Glucocorticoid Receptor Deficiency Attenuates Aging- and HFD-Induced Obesity and Impairs the Feeding-Fasting Transition. Diabetes.

[87] Shen Y., Roh H.C., Kumari M., Rosen E.D. (2017). Adipocyte glucocorticoid receptor is important in lipolysis and insulin resistance due to exogenous steroids, but not insulin resistance caused by high fat feeding. Mol. Metab..

[88] Hayashi R., Okuno Y., Mukai K., Kitamura T., Hayakawa T., Onodera T., Murata M., Fukuhara A., Imamura R., Miyagawa Y. (2019). Adipocyte GR Inhibits Healthy Adipose Expansion Through Multiple Mechanisms in Cushing Syndrome. Endocrinology.

[89] Mousovich-Neto F., Matos M.S., Costa A.C.R., de Melo Reis R.A., Atella G.C., Miranda-Alves L., Carvalho D.P., Ketzer L.A., Correa da Costa V.M. (2019). Brown adipose tissue remodelling induced by corticosterone in male Wistar rats. Exp. Physiol..

[90] Deng J., Guo Y., Yuan F., Chen S., Yin H., Jiang X., Jiao F., Wang F., Ji H., Hu G. (2020). Autophagy inhibition prevents glucocorticoid-increased adiposity via suppressing BAT whitening. Autophagy.

[91] Glantschnig C., Mattijssen F., Vogl E.S., Ali Khan A., Rios Garcia M., Fischer K., Muller T., Uhlenhaut H., Nawroth P., Scheideler M. (2019). The glucocorticoid receptor in brown adipocytes is dispensable for control of energy homeostasis. EMBO Rep..

[92] Poggioli R., Ueta C.B., Drigo R.A., Castillo M., Fonseca T.L., Bianco A.C. (2013). Dexamethasone reduces energy expenditure and increases susceptibility to diet-induced obesity in mice. Obesity (Silver Spring).

[93] Liu J., Kong X., Wang L., Qi H., Di W., Zhang X., Wu L., Chen X., Yu J., Zha J. (2013). Essential roles of 11beta-HSD1 in regulating brown adipocyte function. J. Mol. Endocrinol..

[94] Doig C.L., Fletcher R.S., Morgan S.A., McCabe E.L., Larner D.P., Tomlinson J.W., Stewart P.M., Philp A., Lavery G.G. (2017). 11beta-HSD1 Modulates the Set Point of Brown Adipose Tissue Response to Glucocorticoids in Male Mice. Endocrinology.

[95] Shin H., Ma Y., Chanturiya T., Cao Q., Wang Y., Kadegowda A.K.G., Jackson R., Rumore D., Xue B., Shi H. (2017). Lipolysis in Brown Adipocytes Is Not Essential for Cold-Induced Thermogenesis in Mice. Cell Metab..

[96] Schreiber R., Diwoky C., Schoiswohl G., Feiler U., Wongsiriroj N., Abdellatif M., Kolb D., Hoeks J., Kershaw E.E., Sedej S. (2017). Cold-Induced Thermogenesis Depends on ATGL-Mediated Lipolysis in Cardiac Muscle, but Not Brown Adipose Tissue. Cell Metab..

[97] Chitraju C., Fischer A.W., Farese R.V., Walther T.C. (2020). Lipid Droplets in Brown Adipose Tissue Are Dispensable for Cold-Induced Thermogenesis. Cell Rep..

[98] Luijten I.H.N., Brooks K., Boulet N., Shabalina I.G., Jaiprakash A., Carlsson B., Fischer A.W., Cannon B., Nedergaard J. (2019). Glucocorticoid-Induced Obesity Develops Independently of UCP1. Cell Rep..

[99] Luijten I.H.N., Cannon B., Nedergaard J. (2019). Glucocorticoids and Brown Adipose Tissue: Do glucocorticoids really inhibit thermogenesis?. Mol. Asp. Med..

[100] Barclay J.L., Agada H., Jang C., Ward M., Wetzig N., Ho K.K. (2015). Effects of glucocorticoids on human brown adipocytes. J. Endocrinol..

[101] Ramage L.E., Akyol M., Fletcher A.M., Forsythe J., Nixon M., Carter R.N., van Beek E.J., Morton N.M., Walker B.R., Stimson R.H. (2016). Glucocorticoids Acutely Increase Brown Adipose Tissue Activity in Humans, Revealing Species-Specific Differences in UCP-1 Regulation. Cell Metab..

[102] Urbach V., Verriere V., Grumbach Y., Bousquet J., Harvey B.J. (2006). Rapid anti-secretory effects of glucocorticoids in human airway epithelium. Steroids.

[103] Buttgereit F., Krauss S., Brand M.D. (1997). Methylprednisolone inhibits uptake of Ca2+ and Na+ ions into concanavalin A-stimulated thymocytes. Biochem. J..

[104] Han J.Z., Lin W., Chen Y.Z. (2005). Inhibition of ATP-induced calcium influx in HT4 cells by glucocorticoids: Involvement of protein kinase A. Acta. Pharmacol. Sin..

[105] Steiner A., Vogt E., Locher R., Vetter (1988). Stimulation of the phosphoinositide signalling system as a possible mechanism for glucocorticoid action in blood pressure control. J. Hypertension. Suppl. Off. J. Int. Soc. Hypertens..

[106] Flaherty R.L., Owen M., Fagan-Murphy A., Intabli H., Healy D., Patel A., Allen M.C., Patel B.A., Flint M.S. (2017). Glucocorticoids induce production of reactive oxygen species/reactive nitrogen species and DNA damage through an iNOS mediated pathway in breast cancer. Breast Cancer Res..

[107] Degerman E., Ahmad F., Chung Y.W., Guirguis E., Omar B., Stenson L., Manganiello V. (2011). From PDE3B to the regulation of energy homeostasis. Curr. Opin. Pharmacol..

[108] Kitamura T., Kitamura Y., Kuroda S., Hino Y., Ando M., Kotani K., Konishi H., Matsuzaki H., Kikkawa U., Ogawa W. (1999). Insulin-induced phosphorylation and activation of cyclic nucleotide phosphodiesterase 3B by the serine-threonine kinase Akt. Mol. Cell Biol..

[109] Degerman E., Landstrom T.R., Wijkander J., Holst L.S., Ahmad F., Belfrage P., Manganiello V. (1998). Phosphorylation and activation of hormone-sensitive adipocyte phosphodiesterase type 3B. Methods.

[110] Sztalryd C., Xu G., Dorward H., Tansey J.T., Contreras J.A., Kimmel A.R., Londos C. (2003). Perilipin A is essential for the translocation of hormone-sensitive lipase during lipolytic activation. J. Cell Biol..

[111] Wang H., Hu L., Dalen K., Dorward H., Marcinkiewicz A., Russell D., Gong D., Londos C., Yamaguchi T., Holm C. (2009). Activation of hormone-sensitive lipase requires two steps, protein phosphorylation and binding to the PAT-1 domain of lipid droplet coat proteins. J. Biol. Chem..

[112] Duncan R.E., Ahmadian M., Jaworski K., Sarkadi-Nagy E., Sul H.S. (2007). Regulation of lipolysis in adipocytes. Annu. Rev. Nutr..

[113] Hafezi-Moghadam A., Simoncini T., Yang Z., Limbourg F.P., Plumier J.C., Rebsamen M.C., Hsieh C.M., Chui D.S., Thomas K.L., Prorock A.J. (2002). Acute cardiovascular protective effects of corticosteroids are mediated by non-transcriptional activation of endothelial nitric oxide synthase. Nat. Med..

[114] Gray N.E., Lam L.N., Yang K., Zhou A.Y., Koliwad S., Wang J.C. (2017). Angiopoietin-like 4 (Angptl4) protein is a physiological mediator of intracellular lipolysis in murine adipocytes. J. Biol. Chem..

[115] Singh A.K., Aryal B., Chaube B., Rotllan N., Varela L., Horvath T.L., Suarez Y., Fernandez-Hernando C. (2018). Brown adipose tissue derived ANGPTL4 controls glucose and lipid metabolism and regulates thermogenesis. Mol. Metab..

[116] Wiper-Bergeron N., Wu D., Pope L., Schild-Poulter C., Hache R.J. (2003). Stimulation of preadipocyte differentiation by steroid through targeting of an HDAC1 complex. EMBO J..

[117] Tang Q.Q., Zhang J.W., Daniel Lane M. (2004). Sequential gene promoter interactions by C/EBPbeta, C/EBPalpha, and PPARgamma during adipogenesis. Biochem. Biophys. Res. Commun..

[118] Giroud M., Tsokanos F.F., Caratti G., Kotschi S., Khani S., Jouffe C., Vogl E.S., Irmler M., Glantschnig C., Gil-Lozano M. (2021). HAND2 is a novel obesity-linked adipogenic transcription factor regulated by glucocorticoid signalling. Diabetologia.

